# Supplementary material for: Long Term Findings Concerning the Mental and Physical Condition, Quality of Life and Sexuality after Laparoscopically Assisted Creation of a Neovagina (Modified Vecchietti Technique) in Young MRKHS (Mayer-Rokitansky-Küster-Hauser-Syndrome) Patients
Source: J Clin Med. 2021 Mar 18;10(6):1269. doi: 10.3390/jcm10061269 (PMC8003280; doi:10.3390/jcm10061269)
Supplement: Supplementary file 1 [file jcm-10-01269-s001.pdf]

Journal of
Clinical Medicine

MDPI

Supplementary data

Table 1S: Preoperative source of information (postoperative data)

	TP2	TP3
How did you hear from the therapeutic options at the Women´s university hospital in Tübingen? (multiple selections possible)	*n* = 82	*n* = 56
practicing gynecologist	37 (45 %)	26 (46 %)
gynecologist at hospital	32 (39 %)	20 (36 %)
other affected women	8 (10 %)	8 (14 %)
internet	41 (50 %)	21 (38 %)
TV	5 (6 %)	2 (4 %)
books/ newspapers	0	0
others	3 (4 %)	1 (2 %)
Was your gynecologist able to give sufficient information?	*n* = 77	*n* = 53
No	20 (26 %)	22 (42 %)
Yes, at least some	22 (29 %)	10 (19 %)
Yes, I was well informed	35 (46 %)	21 (40 %)

Table 2S: contact to others

	TP2	TP3
With whom do you talk about the MRKHS diagnosis? (multiple selections possible)	*n* = 82	*n* = 56
mother	62 (76 %)	39 (70 %)
father	35 (43 %)	19 (34 %)
brother (s)	11 (13 %)	7 (13 %)
sister (s)	24 (29 %)	14 (25 %)
partner	41 (50 %)	34 (61 %)
friends	40 (49 %)	28 (50 %)
others (gynecologist, grandmother, aunts, others affected)	18 (22 %)	13 (23 %)
no one	5 (6 %)	3 (5 %)
Do you have contact to others affected/ self-help groups? (multiple selections possible)	*n* = 82	*n* = 54
yes, I contacted others	37 (45 %)	20 (37 %)
yes, I was contacted by others	20 (24 %)	17 (32 %)
No	43 (52 %)	28 (52 %)

NeoCope questionnaire

1. informed consent
2. NeoCope TP1

wird von der Studienleitung abgetrennt

Universitätsklinikum Tübingen
Abteilung für Psychosomatische Medizin und Psychotherapie
und Universitäts-Frauenklinik

Wird von der Studienleitung ausgefüllt:

Patientinnen-Code: _______________

Einverständniserklärung

Vielen Dank für Ihre Bereitschaft, an dieser Studie zum Mayer-Rokitansky-Küster-Hauser-Syndrom (MRKH-Syndrom) teil zu nehmen.

Die Abteilung für Psychosomatische Medizin und Psychotherapie sowie die Universitäts-Frauenklinik Tübingen führen diese Studie gemeinsam durch. Ziel ist es, mehr über die körperliche und psychische Gesundheit betroffener Frauen zu erfahren. Auf dieser Grundlage wird ein psychosomatisches Begleitangebot für MRKH-Patientinnen weiterentwickelt, durch welches die Patientinnen während der operativen Therapie und Nachsorge unterstützt werden.

Ihre Angaben in diesem Fragebogen werden in anonymisierter Form ausschließlich zu wissenschaftlichen Zwecken weiterverarbeitet, so dass niemand die Angaben auf ihre Person zurückführen kann. Die Teilnahme oder Nicht-Teilnahme an dieser Studie hat keinen Einfluss auf die medizinische oder psychosomatische Behandlung und Begleitung Ihrer Person. Sie können zu jedem Zeitpunkt die weitere Teilnahme an dieser Studie beenden, ohne dass Ihnen dadurch Nachteile entstehen.

Bitte füllen Sie diesen Fragebogen selbständig aus und beantworten Sie jede der Fragen entsprechend der jeweiligen Anleitung.

Ich habe die vorliegende Einverständniserklärung gelesen und verstanden.
Ich bin damit einverstanden, dass meine Angaben in der oben beschriebenen Form erhoben und für wissenschaftliche Zwecke in anonymisierter Form weiterverarbeitet werden.

Name: ____

Geburtsdatum: ____

Tübingen, den _______________ ___________________________
Unterschrift

Universitätsklinikum Tübingen
Abteilung für Psychosomatische Medizin und Psychotherapie
und Universitäts-Frauenklinik

NeoCope prä

Fragebogen für Frauen mit MRKH-Syndrom

Bitte füllen Sie diesen Fragebogen selbständig aus.
Bitte antworten Sie auf jede der Fragen, indem Sie
- auf den vorgesehenen Linien schreiben
- das Kästchen bei der auf Sie oder Ihre Situation zutreffenden Aussage eindeutig ankreuzen

Bitte kreuzen Sie bei jeder Frage nur ein Kästchen an. Bei den Fragen, an welchen dies ausdrücklich durch den Zusatztext *„Mehrfachantworten sind möglich!"* vermerkt ist, kreuzen Sie bitte alle Kästchen bei der auf Sie oder Ihre Situation zutreffenden Aussagen an.

Vielen Dank für Ihre Mitarbeit!

1. Wie ist Ihre Körpergröße? _______ cm **2. Wie ist Ihr aktuelles Gewicht?** ________ kg

3. Geburtsjahr: _______________

4. In welchem Land sind Sie geboren? _____________________

5. Aus welchem Land oder welchen Ländern stammen Ihre Eltern?

____

6. Familienstand:
- ❑ ledig
- ❑ geschieden
- ❑ verheiratet
- ❑ verwitwet
- ❑ getrennt

7. Haben Sie derzeit eine feste Partnerschaft?
- ❑ Nein, ich habe keine feste Partnerschaft.
- ❑ Ja, ich habe einen festen Partner.

8. Wohnsituation:
- ❑ Ich wohne bei meinen Eltern.
- ❑ Ich wohne alleine in einer eigenen Wohnung.
- ❑ Ich wohne in einer Wohngemeinschaft.
- ❑ Ich wohne mit meinem Partner in einer gemeinsamen Wohnung.

9. Bitte nennen Sie uns die <u>ersten drei Ziffern</u> der Postleitzahl Ihres Wohnortes:

❑ ❑ ❑

10. Welches ist Ihr höchster Schulabschluss?
- ❑ kein Abschluss
- ❑ (Fach-) Abitur
- ❑ Hauptschulabschluss
- ❑ Hochschulabschluss
- ❑ Realschulabschluss (mittlere Reife)

Universitätsklinikum Tübingen
Abteilung für Psychosomatische Medizin und Psychotherapie
und Universitäts-Frauenklinik

11. Welchen Beruf üben Sie derzeit aus?

❑ arbeitslos ❑ Studentin
❑ Hausfrau ❑ Angestellte
❑ Rentnerin/ Frührentnerin ❑ Beamtin
❑ Schülerin ❑ selbständig
❑ Auszubildende ❑ sonstiges: ______________________________

12. Wie alt waren Sie, als zum ersten Mal auffiel, dass mit Ihrem Körper etwas anders ist?

Alter in Jahren: ____________ und _______ Monate

13. Aus welchem Anlass erfolgte die Untersuchung, die zur Diagnose des MRKH-Syndroms geführt hat?

Mehrfachantworten sind möglich!

❑ Regelblutung blieb aus
❑ erfolgloser Geschlechtsverkehr
❑ Petting (Partner hat es beim Streicheln bemerkt)
❑ Vorsorgeuntersuchung beim Arzt
❑ selbst getastet (z.B. beim „Selbst-Streicheln" = Masturbation)
❑ fiel durch die Untersuchung anderer Fehlbildungen (z.B. Skelett, Niere, ...) auf❑
❑ auf Drängen der Mutter

❑ anderer: ______________________________

14. Welche Untersuchungen wurden zur Diagnose des MRKH-Syndroms durchgeführt?

Mehrfachantworten sind möglich!

❑ Tastuntersuchung beim Frauenarzt ❑ Blutuntersuchung (Hormonwerte)
❑ Ultraschall ❑ Bauchspiegelung (Laparaskopie)
❑ Computertomographie (CT) / Magnetresonanztomographie (MRT) („Röhre) ❑ Tastuntersuchung in Narkose
❑ andere: ______________________________

15. Wie alt waren Sie, als die richtige Diagnose MRKH-Syndrom zum ersten Mal gestellt wurde?

Alter in Jahren: ____________ und _______ Monate

16. Durch wen wurde die Diagnose MRKH-Syndrom gestellt?

Bitte kreuzen Sie die Person an, welche die richtige Diagnose als erste gestellt hat.

❑ Hausarzt
❑ Frauenarzt
❑ Arzt in Fachklinik

❑ andere: ______________________________

17. Was fühlten Sie, als Ihnen die Diagnose mitgeteilt wurde?

❑ Verzweiflung
❑ nicht-wahr-haben-wollen
❑ ein Gefühl, wegen nicht vorhandener Scheide keine richtige Frau zu sein (Insuffizienzgefühl)
❑ Minderwertigkeitsgefühle/ Schock wegen Kinderlosigkeit
❑ Sorge, keinen Partner zu finden

❑ sonstiges: ______________________________

Seite 2

Universitätsklinikum Tübingen
Abteilung für Psychosomatische Medizin und Psychotherapie
und Universitäts-Frauenklinik

18. Wie stark belastet Sie die Diagnose des MRKH-Syndroms?

sehr wenig 1	2	3	4	5	6	7	8	9	sehr stark 10

19. Liegen bei Ihnen weitere Fehlbildungen vor?
Mehrfachantworten sind möglich!
❑ Nieren/ Harnwege
❑ Herzfehler
❑ Hörschaden
❑ Wirbelsäule/ Skelett (auch Finger, Zehen, Zähne/ Kiefer)
❑ Muskelschwäche
❑ ich wurde als Kind wegen eines Leistenbruchs operiert

❑ andere: ______________________________
❑ keine weiteren Fehlbildungen

20. Wie viele Geschwister haben Sie?

_______ Schwestern, ________ Brüder

21. Haben Sie zyklische Beschwerden (d.h. regelmäßige monatliche Beschwerden über 1-3 Tage)?
Mehrfachantworten sind möglich!
❑ Brustspannen
❑ Mittelschmerz (Ziehen im Unterbauch)
❑ Reizbarkeit
❑ Ausfluss
❑ Heißhunger
❑ geblähter Bauch

❑ andere: ______________________________
❑ keine Beschwerden

22. Welche Themen beschäftigen Sie im Zusammenhang mit MRKH am meisten?
Mehrfachantworten sind möglich!
❑ die ungewollte Kinderlosigkeit
❑ die Therapie/ Operation
❑ eine erfüllte Partnerschaft führen
❑ ein erfülltes Sexualleben haben
❑ mein Selbstwertgefühl
❑ die Beziehung zu meiner Mutter
❑ körperlich anders zu sein als meine Freundinnen
❑ ich fühle mich anders als andere Frauen

❑ sonstige: ______________________________

23. Haben Sie derzeit Sexualkontakt?
❑ Nein
❑ Ja: ______________ mal pro Woche oder ______________ pro Monat
❑ Petting
❑ Geschlechtsverkehr
❑ beides

24. Wie zufrieden sind Sie mit dem Sexualkontakt?

sehr unzufrieden 1	2	3	4	5	6	7	8	9	sehr zufrieden 10

Dankeschön.

Seite 3

3. NeoCope TP2

Universitätsklinikum Tübingen
Abteilung für Psychosomatische Medizin und Psychotherapie
und Universitäts-Frauenklinik

NeoCope post 6M

Fragebogen für Frauen mit MRKH-Syndrom

Bitte füllen Sie diesen Fragebogen selbständig aus.
Bitte antworten Sie auf jede der Fragen, indem Sie
- auf den vorgesehenen Linien schreiben
- das Kästchen bei der auf Sie oder Ihre Situation zutreffenden Aussage eindeutig ankreuzen

Bitte kreuzen Sie bei jeder Frage nur ein Kästchen an. Bei den Fragen, an welchen dies ausdrücklich durch den Zusatztext *„Mehrfachantworten sind möglich!"* vermerkt ist, kreuzen Sie bitte alle Kästchen bei der auf Sie oder Ihre Situation zutreffenden Aussagen an.

Vielen Dank für Ihre Mitarbeit!

1. Wie ist Ihre Körpergröße? _______ cm **2. Wie ist Ihr aktuelles Gewicht?** ________ kg

3. Geburtsjahr: _______________

6. Familienstand:
❑ ledig ❑ geschieden
❑ verheiratet ❑ verwitwet
❑ getrennt

7. Haben Sie derzeit eine feste Partnerschaft?
❑ Nein, ich habe keine feste Partnerschaft.
❑ Ja, ich habe einen festen Partner.

8. Wohnsituation:
❑ Ich wohne bei meinen Eltern.
❑ Ich wohne alleine in einer eigenen Wohnung.
❑ Ich wohne in einer Wohngemeinschaft.
❑ Ich wohne mit meinem Partner in einer gemeinsamen Wohnung.

10. Welches ist Ihr höchster Schulabschluss?
❑ kein Abschluss ❑ (Fach-) Abitur
❑ Hauptschulabschluss ❑ Hochschulabschluss
❑ Realschulabschluss (mittlere Reife)

11. Welchen Beruf üben Sie derzeit aus?
❑ arbeitslos ❑ Studentin
❑ Hausfrau ❑ Angestellte
❑ Rentnerin/ Frührentnerin ❑ Beamtin
❑ Schülerin ❑ selbständig
❑ Auszubildende ❑ sonstiges: ______________________________

Seite 1

Universitätsklinikum Tübingen
Abteilung für Psychosomatische Medizin und Psychotherapie
und Universitäts-Frauenklinik

17. Was fühlen Sie hinsichtlich der MRKH-Diagnose nach der Operation?
❑ Verzweiflung
❑ nicht-wahr-haben-wollen
❑ ein Gefühl, wegen nicht vorhandener Scheide keine richtige Frau zu sein
❑ Minderwertigkeitsgefühle/ Schock wegen Kinderlosigkeit
❑ Sorge, keinen Partner zu finden

❑ sonstiges: ____

18. Wie stark belastet Sie die Diagnose des MRKH-Syndroms?

sehr wenig									sehr stark
1	2	3	4	5	6	7	8	9	10

21. Haben Sie zyklische Beschwerden (d.h. regelmäßige monatliche Beschwerden über 1 - 3 Tage)?
Mehrfachantworten sind möglich!
❑ Brustspannen
❑ Mittelschmerz (Ziehen im Unterbauch)
❑ Reizbarkeit
❑ Ausfluss
❑ Heißhunger
❑ geblähter Bauch

❑ andere: ____
❑ keine Beschwerden

25. Wie lange haben Sie zwischen Diagnose und Operation gewartet?

Wartezeit in Jahren: _________ und _______ Monate

26. Haben Sie versucht, <u>vor</u> der Operation das Scheidengrübchen mit einem Phantom/ Konus vorzudehnen?
❑ Nein
❑ Ja: ca. _______ Stunden pro Tag über _____ Wochen

27. War die Information der behandelnden Frauenklinik zur Behandlung ausreichend?
❑ Nein
❑ Ja, ich wusste genau Bescheid
Anmerkungen:

28. Was waren oder sind Ihre Gründe für die Operation?
Mehrfachantworten sind möglich!
❑ Wunsch nach Vollwertigkeit als Frau
❑ Wunsch nach Geschlechtsverkehr
❑ Hoffnung auf Orgasmus
❑ Wunsch vom Geschlechtspartner genauso gesehen zu werden wie andere Frauen
❑ auf Drängen des Vaters
❑ auf Drängen anderer Angehöriger
❑ auf Drängen des Partners
❑ auf Drängen der Mutter
❑ sonstige: ____

29. Wie zufrieden sind Sie mit dem Ergebnis der Operation?

überhaupt nicht zufrieden									sehr zufrieden
1	2	3	4	5	6	7	8	9	10

Seite 2

Universitätsklinikum Tübingen
Abteilung für Psychosomatische Medizin und Psychotherapie
und Universitäts-Frauenklinik

30. Wie gut haben Sie sich in der Klinik betreut gefühlt?

sehr schlecht … sehr gut

1	2	3	4	5	6	7	8	9	10

Anmerkungen zum Klinikaufenthalt:

31. Wie stark beurteilen Sie rückblickend die Schmerzen, die sie während der Zeit auf der Wachstation erlitten haben?

keine Schmerzen … sehr starke Schmerzen

0 1 2 3 4 5 6 7 8 9 10

32. Würden Sie sich rückblickend noch einmal für die Operation entscheiden?

❑ Nein, weil: ____________________

❑ Ja, weil: ____________________

33. Würden Sie die Behandlung anderen betroffenen Frauen empfehlen?

❑ Nein, weil: ____________________

❑ Ja, weil: ____________________

34. Wie oft und wie lange haben Sie den Vaginaldilatator (Phantom, Konus) benutzt?

im 1. Monat __________ h/Tag, jeden ______ Tag

im 2.-3. Monat __________ h/Tag, jeden ______ Tag

im 3.-6. Monat __________ h/Tag, jeden ______ Tag

aktuell __________ h/Tag, jeden ______ Tag

ab __________ Wochen nach der Operation wurde das Phantom nicht mehr benutzt

35. Haben oder hatten Sie Schmerzen bei der Verwendung des Vaginaldilatators (Phantoms, Konus)?

❑ Nein ❑ Ja, manchmal
❑ Ja, bei den ersten Malen ❑ Ja, immer

36. Kam es durch die Verwendung des Vaginaldilatators (Phantoms, Konus) zu einer Blutung?

❑ Nein ❑ Ja, manchmal
❑ Ja, bei den ersten Malen ❑ Ja, immer

37. Haben Sie das Miederhöschen getragen und wie lange?

❑ Nein
❑ Ja, die ersten ________ Monate für ________ Stunden

Seite 3

Universitätsklinikum Tübingen
Abteilung für Psychosomatische Medizin und Psychotherapie
und Universitäts-Frauenklinik

38. Wie lange haben Sie die Östrogen-Creme (Ovestin) verwendet?

____________________ Monate

39. Hatten Sie schon einmal Geschlechtsverkehr?
❑ Nein (weiter mit Frage 44)
❑ Ja

40. Wann hatten Sie nach der Operation den <u>ersten</u> Geschlechtsverkehr?
❑ Ich hatte noch keinen Geschlechtsverkehr nach der Operation (weiter mit Frage 36).
❑ nach ______ Wochen oder _______ Tagen

40.1 Ab wann hatten Sie <u>regelmäßig</u> Geschlechtsverkehr?
________ Wochen nach der Operation

40.2 Wie häufig?
________ mal pro Woche oder ________mal pro Monat

41. Haben Sie <u>derzeit</u> Geschlechtsverkehr?
❑ Nein
❑ Ja: _______ mal pro Woche oder ________mal pro Monat

42. Wie zufrieden sind Sie mit dem Geschlechtsverkehr?

sehr unzufrieden									sehr zufrieden
1	2	3	4	5	6	7	8	9	10

43. Hatten Sie seit der Operation mehrere Geschlechtspartner?
❑ Nein
❑ Ja – Anzahl: ________

44. Wie bekommen Sie einen Orgasmus?
Mehrfachantworten sind möglich!
❑ (Tag-) Träume
❑ Selbst-Streicheln (Masturbation)
❑ Streicheln durch Partner (Petting)
❑ Kohabitation (Geschlechtsverkehr) <u>ohne</u> Streicheln
❑ Kohabitation (Geschlechtsverkehr) <u>mit</u> Streicheln
❑ ich bekomme keinen Orgasmus

22. Welche Themen beschäftigen Sie im Zusammenhang mit MRKH am meisten?
Mehrfachantworten sind möglich!
❑ die ungewollte Kinderlosigkeit
❑ die Therapie/ Operation
❑ eine erfüllte Partnerschaft führen
❑ ein erfülltes Sexualleben haben
❑ mein Selbstwertgefühl
❑ die Beziehung zu meiner Mutter
❑ körperlich anders zu sein als meine Freundinnen
❑ ich fühle mich anders als andere Frauen

❑ sonstige: ____

Universitätsklinikum Tübingen
Abteilung für Psychosomatische Medizin und Psychotherapie
und Universitäts-Frauenklinik

45. Wie stark belastet Sie die gleichzeitige Diagnose der Unfruchtbarkeit (Infertilität, kein Kind austragen zu können)?

sehr wenig 1	2	3	4	5	6	7	8	9	sehr stark 10

46. Hat sich Ihr Verhältnis gegenüber Ihrer Mutter seit der Operation verändert?
❑ Nein, es ist unverändert.
❑ Ja, es hat sich verbessert/ ist offener geworden.
❑ Ja, ich fühle mich ihr gegenüber unabhängiger.
❑ Ja, aber es hat sich verschlechtert.

❑ Ja: ____

47. Wie gut schätzen Sie Ihr Verhältnis gegenüber Ihrer Mutter zum heutigen Zeitpunkt ein?

sehr schlecht 1	2	3	4	5	6	7	8	9	sehr gut 10

48. Was glauben Sie, wie stark Ihre Mutter durch die Diagnose des MRKH-Syndroms bei Ihnen belastet ist?

sehr wenig 1	2	3	4	5	6	7	8	9	sehr stark 10

49. Hat sich Ihr Gefühl bzw. Ihr Auftreten gegenüber Männern nach der Operation im Vergleich zu vor der Operation verändert?
❑ Nein.
❑ Ja, ich bin jetzt selbstbewusster.
❑ Ja, ich kann als Frau jetzt besser auf Männer zugehen.

❑ Ja:____

50. Mit wem sprechen Sie über die MRKH-Diagnose?
Mehrfachantworten sind möglich!
❑ Mutter ❑ Partner
❑ Vater ❑ Freunde
❑ Brüder ❑ andere: ____________________________
❑ Schwestern ❑ mit niemandem

51. Sind/ waren Sie in psychosomatischer oder psychotherapeutischer Betreuung?
Mehrfachantworten sind möglich!
❑ Nein
❑ Ja, stationär in einer Klinik
❑ Ja, in ambulanter Psychotherapie
❑ Ja, im Rahmen der Neovagina-Operation an der Universitäts-Frauenklinik Tübingen

Universitätsklinikum Tübingen
Abteilung für Psychosomatische Medizin und Psychotherapie
und Universitäts-Frauenklinik

52. Wenn Sie an der Universitäts-Frauenklinik psychosomatisch begleitet wurden: Wie sehr hat Ihnen dieses Angebot geholfen?

sehr wenig									sehr viel
1	2	3	4	5	6	7	8	9	10

❑ kein psychosomatisches Begleitangebot

53. Haben Sie Kontakt zu anderen betroffenen Frauen/ zu Selbsthilfegruppen?
Mehrfachantworten sind möglich!
❑ Ja, ich habe andere kontaktiert
❑ Ja, ich wurde von anderen kontaktiert
❑ Nein

54. Falls Sie Kontakt haben: Empfanden Sie den Kontakt zu anderen betroffenen Frauen als hilfreich?
❑ Nein
❑ Ja
Anmerkungen:

55. Falls Sie keinen Kontakt haben: Hätten Sie sich Kontakt zu anderen betroffenen Frauen oder Selbsthilfegruppen gewünscht?
❑ Nein
❑ Ja
Anmerkungen

56. Wie haben Sie von den Therapiemöglichkeiten an der Universitäts-Frauenklinik erfahren?
Mehrfachantworten sind möglich!
❑ Frauenarzt ❑ Internet
❑ Arzt in Fachklinik ❑ Fernsehbeitrag
❑ andere betroffene Frauen ❑ Bücher / Zeitschriften

❑ andere: ____________________

57. Konnte Ihnen Ihr Frauenarzt die notwendigen Informationen geben?
❑ Nein. Folgende Informationen haben gefehlt: ____________________

❑ Ja, teilweise. Gefehlt hat: ____________________

❑ Ja, wusste genau Bescheid.

Dankeschön.

Seite 6

4. NeoCope TP3

Universitätsklinikum Tübingen
Abteilung für Psychosomatische Medizin und Psychotherapie
und Universitäts-Frauenklinik

NeoCope post 12M

Fragebogen für Frauen mit MRKH-Syndrom

Bitte füllen Sie diesen Fragebogen selbständig aus.
Bitte antworten Sie auf jede der Fragen, indem Sie
- auf den vorgesehenen Linien schreiben
- das Kästchen bei der auf Sie oder Ihre Situation zutreffenden Aussage eindeutig ankreuzen

Bitte kreuzen Sie bei jeder Frage nur ein Kästchen an. Bei den Fragen, an welchen dies ausdrücklich durch den Zusatztext *„Mehrfachantworten sind möglich!“* vermerkt ist, kreuzen Sie bitte alle Kästchen bei der auf Sie oder Ihre Situation zutreffenden Aussagen an.

Vielen Dank für Ihre Mitarbeit!

1. Wie ist Ihre Körpergröße? ________ cm **2. Wie ist Ihr aktuelles Gewicht?** _________ kg

6. Familienstand:
❑ ledig ❑ geschieden
❑ verheiratet ❑ verwitwet
❑ getrennt

7. Haben Sie derzeit eine feste Partnerschaft?
❑ Nein, ich habe keine feste Partnerschaft.
❑ Ja, ich habe einen festen Partner.

8. Wohnsituation:
❑ Ich wohne bei meinen Eltern.
❑ Ich wohne alleine in einer eigenen Wohnung.
❑ Ich wohne in einer Wohngemeinschaft.
❑ Ich wohne mit meinem Partner in einer gemeinsamen Wohnung.

10. Welches ist Ihr höchster Schulabschluss?
❑ kein Abschluss ❑ (Fach-) Abitur
❑ Hauptschulabschluss ❑ Hochschulabschluss
❑ Realschulabschluss (mittlere Reife)

11. Welchen Beruf üben Sie derzeit aus?
❑ arbeitslos ❑ Studentin
❑ Hausfrau ❑ Angestellte
❑ Rentnerin/ Frührentnerin ❑ Beamtin
❑ Schülerin ❑ selbständig
❑ Auszubildende ❑ sonstiges: ______________________________

18. Wie stark belastet Sie die Diagnose des MRKH-Syndroms?

sehr wenig 1	2	3	4	5	6	7	8	9	sehr stark 10

Seite 1

Universitätsklinikum Tübingen
Abteilung für Psychosomatische Medizin und Psychotherapie
und Universitäts-Frauenklinik

21. Haben Sie zyklische Beschwerden (d.h. regelmäßige monatliche Beschwerden über 1-3 Tage)?

Mehrfachantworten sind möglich!

❑ Brustspannen ❑ Ausfluss
❑ Mittelschmerz (Ziehen im Unterbauch) ❑ Heißhunger
❑ Reizbarkeit ❑ geblähter Bauch

❑ andere: ____
❑ keine Beschwerden

29. Wie zufrieden sind Sie mit dem Ergebnis der Operation?

überhaupt nicht zufrieden									sehr zufrieden
1	2	3	4	5	6	7	8	9	10

30. Wie gut haben Sie sich in der Klinik betreut gefühlt?

sehr schlecht									sehr gut
1	2	3	4	5	6	7	8	9	10

Anmerkungen zum Klinikaufenthalt:

34. Wie oft und wie lange haben Sie den Vaginaldilatator (Phantom, Konus) benutzt?

im 1. Monat __________ h/Tag, jeden ______ Tag

im 2.-3. Monat ________ h/Tag, jeden ______ Tag

im 3.-6. Monat ________ h/Tag, jeden ______ Tag

aktuell ________ h/Tag, jeden ______ Tag

ab _________ Wochen nach der Operation wurde das Phantom nicht mehr benutzt

35. Haben oder hatten Sie Schmerzen bei der Verwendung des Vaginaldilatators (Phantoms, Konus)?

❑ Nein ❑ Ja, manchmal
❑ Ja, bei den ersten Malen ❑ Ja, immer

36. Kam es durch die Verwendung des Vaginaldilatators (Phantoms, Konus) zu einer Blutung?

❑ Nein ❑ Ja, manchmal
❑ Ja, bei den ersten Malen ❑ Ja, immer

37. Haben Sie das Miederhöschen getragen und wie lange?

❑ Nein
❑ Ja, die ersten _______ Monate für _______ Stunden

38. Wie lange haben Sie die Östrogen-Creme (Ovestin) verwendet?

____________________ Monate

Universitätsklinikum Tübingen
Abteilung für Psychosomatische Medizin und Psychotherapie
und Universitäts-Frauenklinik

39. Hatten Sie schon einmal Geschlechtsverkehr?
❑ Nein (weiter mit Frage 36)
❑ Ja

40. Wann hatten Sie nach der Operation den ersten Geschlechtsverkehr?
❑ Ich hatte noch keinen Geschlechtsverkehr nach der Operation (weiter mit Frage 36).
❑ nach ______ Wochen oder _______ Tagen

40.1 Ab wann hatten Sie regelmäßig Geschlechtsverkehr?
________ Wochen nach der Operation

40.2 Wie häufig?
________ mal pro Woche oder ________mal pro Monat

41. Haben Sie derzeit Geschlechtsverkehr?
❑ Nein
❑ Ja: _______ mal pro Woche oder ________mal pro Monat

42. Wie zufrieden sind Sie mit dem Geschlechtsverkehr?

sehr unzufrieden — sehr zufrieden

1	2	3	4	5	6	7	8	9	10

43. Hatten Sie seit der Operation mehrere Geschlechtspartner?
❑ Nein
❑ Ja – Anzahl: ________

44. Wie bekommen Sie einen Orgasmus?
Mehrfachantworten sind möglich!
❑ (Tag-) Träume
❑ Selbst-Streicheln (Masturbation)
❑ Streicheln durch Partner (Petting)
❑ Kohabitation (Geschlechtsverkehr) ohne Streicheln
❑ Kohabitation (Geschlechtsverkehr) mit Streicheln
❑ ich bekomme keinen Orgasmus

22. Welche Themen beschäftigen Sie im Zusammenhang mit MRKH am meisten?
Mehrfachantworten sind möglich!
❑ die ungewollte Kinderlosigkeit
❑ die Therapie/ Operation
❑ eine erfüllte Partnerschaft führen
❑ ein erfülltes Sexualleben haben
❑ mein Selbstwertgefühl
❑ die Beziehung zu meiner Mutter
❑ körperlich anders zu sein als meine Freundinnen
❑ ich fühle mich anders als andere Frauen

❑ sonstige: ____

45. Wie stark belastet Sie die gleichzeitige Diagnose der Unfruchtbarkeit (Infertilität, kein Kind austragen zu können)?

sehr wenig — sehr stark

1	2	3	4	5	6	7	8	9	10

Universitätsklinikum Tübingen
Abteilung für Psychosomatische Medizin und Psychotherapie
und Universitäts-Frauenklinik

46. Hat sich Ihr Verhältnis gegenüber Ihrer Mutter seit der Operation verändert?
❑ Nein, es ist unverändert.
❑ Ja, es hat sich verbessert/ ist offener geworden.
❑ Ja, ich fühle mich ihr gegenüber unabhängiger.
❑ Ja, aber es hat sich verschlechtert.

❑ Ja: ____________________

47. Wie gut schätzen Sie Ihr Verhältnis gegenüber Ihrer Mutter zum heutigen Zeitpunkt ein?

sehr schlecht									sehr gut
1	2	3	4	5	6	7	8	9	10

48. Was glauben Sie, wie stark Ihre Mutter durch die Diagnose des MRKH-Syndroms bei Ihnen belastet ist?

sehr wenig									sehr stark
1	2	3	4	5	6	7	8	9	10

49. Hat sich Ihr Gefühl bzw. Ihr Auftreten gegenüber Männern nach der Operation im Vergleich zu vor der Operation verändert?
❑ Nein.
❑ Ja, ich bin jetzt selbstbewusster.
❑ Ja, ich kann als Frau jetzt besser auf Männer zugehen.

❑ Ja: ____________________

50. Mit wem sprechen Sie über die MRKH-Diagnose?
Mehrfachantworten sind möglich!
❑ Mutter ❑ Partner
❑ Vater ❑ Freunde
❑ Brüder ❑ andere: ____________________
❑ Schwestern ❑ mit niemandem

51. Sind/ waren Sie in psychosomatischer oder psychotherapeutischer Betreuung?
Mehrfachantworten sind möglich!
❑ Nein
❑ Ja, stationär in einer Klinik
❑ Ja, in ambulanter Psychotherapie
❑ Ja, im Rahmen der Neovagina-Operation an der Universitäts-Frauenklinik Tübingen

52. Wenn Sie an der Universitäts-Frauenklinik psychosomatisch begleitet wurden: Wie sehr hat Ihnen dieses Angebot geholfen?

sehr wenig									sehr viel
1	2	3	4	5	6	7	8	9	10

❑ kein psychosomatisches Begleitangebot

Seite 4

Universitätsklinikum Tübingen
Abteilung für Psychosomatische Medizin und Psychotherapie
und Universitäts-Frauenklinik

53. Haben Sie Kontakt zu anderen betroffenen Frauen/ zu Selbsthilfegruppen?
Mehrfachantworten sind möglich!
❑ Ja, ich habe andere kontaktiert
❑ Ja, ich wurde von anderen kontaktiert
❑ Nein

54. Falls Sie Kontakt haben: Empfanden Sie den Kontakt zu anderen betroffenen Frauen als hilfreich?
❑ Nein
❑ Ja
Anmerkungen:

55. Falls Sie keinen Kontakt haben: Hätten Sie sich Kontakt zu anderen betroffenen Frauen oder Selbsthilfegruppen gewünscht?
❑ Nein
❑ Ja
Anmerkungen

56. Wie haben Sie von den Therapiemöglichkeiten an der Universitäts-Frauenklinik erfahren?
Mehrfachantworten sind möglich!
❑ Frauenarzt ❑ Internet
❑ Arzt in Fachklinik ❑ Fernsehbeitrag
❑ andere betroffene Frauen ❑ Bücher / Zeitschriften

❑ andere: ____________________

57. Konnte Ihnen Ihr Frauenarzt die notwendigen Informationen geben?
❑ Nein. Folgende Informationen haben gefehlt: ____________________

❑ Ja, teilweise. Gefehlt hat: ____________________

❑ Ja, wusste genau Bescheid.

Dankeschön.
